# Factors influencing medical students career intentions in Flanders, Estonia, and Hungary: a multivariable analysis

**DOI:** 10.3399/BJGPO.2024.0087

**Published:** 2025-05-07

**Authors:** Marta Velgan, Peter Vajer, Nele R Michels, Mart Einasto, Ruth Kalda

**Affiliations:** 1 Institute of Family Medicine and Public Health, University of Tartu, Tartu, Estonia; 2 Department of Family Medicine, Semmelweis University, Budapest, Hungary; 3 The Interuniversity Centre of General Practice Education, Leuven, Belgium; 4 School of Governance, Law and Society, Tallinn University, Tallinn, Estonia

**Keywords:** undergraduate education, postgraduate education, students, medical, workforce, general practice

## Abstract

**Background:**

The career decisions of medical students are pivotal in shaping the future healthcare workforce. In many countries, the number of medical students who choose general practice as their career is insufficient to meet the needs of the healthcare system.

**Aim:**

To describe the factors influencing medical students’ career intentions and their preference for a career in general practice.

**Design & setting:**

A cross-sectional study involving medical students from Flanders (Belgium), Estonia, and Hungary.

**Method:**

An online questionnaire, which was sent to undergraduate medical students, was used to gather data. Multivariable logistic regression was conducted.

**Results:**

Altogether 1601 medical students participated in this study, which found that 18.5% of the participants were interested in general practice. Factors related to medical students and the curriculum that predicted the interest in general practice were being a woman, being a medical student from Flanders, being a sixth-year medical student, coming from a rural area, and having GP role models. Students preferring general practice named the following factors as important: short and low-intensity training programme; having long-term and close relationship with patients; continuity of care; regular and flexible working hours; and opportunities to achieve work–life balance.

**Conclusion:**

This study adds further evidence of which characteristics and factors can predict medical students’ interest in general practice; having GP role models being the most important predictor. Further research into which qualities medical students value in their role models could give us better understanding on how we can support GPs to be better advocates for their specialty and thereby help increase interest in general practice.

## How this fits in

Medical students’ low interest in general practice is one of the many reasons for GP shortages. Many studies that explore the factors influencing medical students’ career intentions have focused on one country or a region. In this study the medical students’ career intentions and factors influencing their choices were explored simultaneously in Flanders (Belgium), Estonia, and Hungary. Various factors predicting the medical students’ interest in a career in general practice were identified; having GP role models being the most important predictor. Further research into the influence of role models on students’ career intentions could give us insight on how GPs could be supported to be better advocates for their specialty.

## Introduction

Choosing a future specialty is one of the most critical decisions that medical students will make at the end of their undergraduate studies. Medical students’ choice of career has an effect on future healthcare workforce composition. A sufficient number and mix of healthcare specialists is needed to ensure effective access to health care.^1^ Although according to the Organisation for Economic Co-operation and Development (OECD) the number of doctors per capita has increased in all OECD countries between 2011 and 2021, in 2021 GPs represented on average less than one-quarter of all doctors.^2^ Although there is a lack of data on the proportion of GPs needed to ensure adequate availability of primary care services, it is well established that the stronger the primary care, the greater benefit for the population. Primary care, provided by family physicians or GPs, is considered to be the foundation of the healthcare system by providing accessible and good functioning health care. A shortage of GPs and limited access to primary care can lead to an increase of waiting time and unmet healthcare needs, and thereby an unnecessary, expensive use of hospital care and emergency departments.^3^

One of the reasons for this shortage is medical students’ low interest in general practice. Previously conducted studies have found that specialties, such as surgery and paediatrics, are more favoured than general practice.^4,5^ Multiple studies have attempted to clarify the decision-making process of medical students and have identified different factors that influence students’ career intentions. These factors can be divided into the following three groups: factors related to students;^6–10^ factors related to the specialty;^11,12^ and factors related to the curriculum.^12,13^ Other studies have described that the medical students’ career intentions can also be influenced by their assessment of their skills and attributes, and how it fits in with the requirements of a specific specialty.^14–16^ Factors that have been shown to increase medical students’ interest in general practice are frequent exposure to general practice during medical studies, the existence of a compulsory GP internship, the size and commitment of the department of general practice, and the existence of positive GP role models.^6,7,14^ Student characteristics that have been previously associated with interest in general practice are being female, older, and married; having a broad undergraduate background and having non-physician parents.^17^ Others have found that medical students prefer general practice for its variety, continuity of care, and work–life balance.^18^ With recent studies it has been described that overall the quality of life and flexibility at work^9–12,19^ have become more important compared with remuneration when choosing a future career.^9,11–13^

The aim of this study was to explore factors influencing medical students’ career intentions simultaneously in Flanders (Belgium), Estonia, and Hungary and create models to describe students who prefer a career in general practice. Existing studies focus on the career intentions of medical students in one country, which is why the aim of including several countries was to see how differences in cultural background, health care, and primary care organisation can affect students’ career intentions. This provided knowledge can deepen our understanding of medical students’ career intentions and help develop strategies to increase their interest in general practice.

## Method

### Study design and setting

To study medical students’ career intentions, a cross-sectional mixed-methods multi-country study was conducted.

### Participants and recruitment

Using SurveyMonkey, a multi-purpose online questionnaire was sent using official universities’ mailing lists to undergraduate medical students in all medical faculties in Flanders, Belgium (University of Antwerp, KU Leuven, the Vrije Universiteit Brussel, Ghent University), Estonia (University of Tartu), and Hungary (Semmelweis University, University of Pécs, University of Debrecen, University of Szeged). In Estonia the online survey was available during February 2020. In April 2020 the survey was sent to students in Belgium. Owing to a low response rate, the questionnaire was reopened from October until December 2020. From June until October 2020 the data were collected from the Hungarian medical students. In Estonia the questionnaire was closed after more than one-third of medical students answered the questionnaire. In Belgium and Hungary the questionnaire was held open longer owing to lower response rate.

### Data collection

A questionnaire was used to collect data. The existing questionnaire was developed based on previous studies and two formerly used questionnaires in medical schools of the Netherlands.^20^ The questionnaire was reviewed by an expert panel (MV, PV, NM, RK) and a pilot study was conducted in Flanders, Estonia, and Hungary in 2018.^21^ The questionnaire was reviewed again after the pilot study. The questionnaire was divided into the following four parts: (1) questions regarding demographics and general information; (2) medical students’ choice of specialty; (3) factors influencing specialty choice; and (4) questions regarding general practice and students’ interest in a career in general practice. Participants could choose from more than 40 different specialties, which are available in all three countries. Afterwards these specialties were gathered into seven groups: general practice or family medicine; internal medicine; emergency medicine and anaesthesiology; paediatrics; surgical specialties; obstetrics and gynaecology; and other (Supplementary Box 1). The participants were asked to choose their first and second choice of specialty, since in Flanders, Estonia, and Hungary new physicians have this option when applying for postgraduate training. In the last two parts, the majority of the questions consisted of statements that were measured on a five-point Likert scale. See Supplementary Box 2 for the English version of this questionnaire.

### Data analysis

Statistical analysis was conducted using IBM SPSS Statistics (version 29.0.0.0). For most part of the analysis medical students’ first and second choice were combined to create two groups: students who prefer general practice (as first and/or second choice) and those who prefer other specialties (as first and/or second choice). Mann–Whitney U-tests and χ^2^ tests were used to test individual impact of different factors between two groups. Multivariate logistic regression analysis was used to create models to describe medical students who prefer general practice. For logistic regression analysis, a purposeful selection process was used. In the regression analyses, we included independent variables that were significantly different between two groups.^22^ In the first regression model, the influence of variables frequently found to be relevant in earlier studies were examined: sex, age, study year, origin, relatives working in medicine, previous education, internship experience at GP office, having GP role models. In the second regression model, the importance of factors related to different specialties, which were divided into six groups, were tested: postgraduate training; scope of practice: general practice or hospital specialties; preferred working conditions; prestige or success orientation; influence to choose a certain specialty (Supplementary Box 3). Lastly, we tried to combine different models to find the best models that describe medical students who prefer general practice. Statistical significance was assumed at *P*<0.05.

## Results

### Characteristics of the participants

In total, 1601 medical students completed the questionnaire of which 764 (47.7%) were from Flanders, 214 (13.4%) from Estonia, and 623 (38.9%) from Hungary. At this moment, approximately 6300 students were studying medicine in Flanders, 900 students in Estonia, and 7700 in Hungary. The characteristics of the participants are presented in Supplementary Table 1.

### Students’ specialty choices

The participants’ specialty choices (first and second choice) are presented in Supplementary Table 2. This table also describes specialty choice based on sex and country of origin. The preferred specialty choices of the participants were surgical specialties, internal medicine and paediatric specialties, emergency medicine and anaesthesiology. Surgical specialties were the preferred first choice and internal medicine specialties the preferred second choice of specialty. Men were more interested in surgical specialties, emergency medicine, and anaesthesiology compared with paediatric specialties, GP, gynaecology and obstetrics. In this study, 18.5% (*n* = 296) of medical students are considering choosing general practice as their first or second choice.

### Factors influencing students’ career intentions

First, the independent factors related to students (age, level of study, sex, country of origin, being from a rural area, having relatives working in medicine, previous higher education) and curriculum (completed internship in general practice and having GP role models) were tested. In model 1 independent factors predicting the preference to general practice were being a woman, being from Flanders, being a sixth-year medical student, and having GP role models.

Second, the factors related to different specialties were tested. The results of group comparisons between students who prefer general practice and those who prefer other specialties are shown in Supplementary Table 3. In model 2 the factors grouped under ‘postgraduate training’, ‘scope of practice: GP’, and ‘preferred working conditions’ turned out to be more important to medical students preferring general practice, compared with factors grouped under ‘scope of practice: hospital specialties’ and ‘prestige or success orientation’. Medical students who prefer a career in general practice consider it important that there are enough postgraduate training placements near where they live. They also favour postgraduate training without complex admission requirements, with shorter duration and a lower-intensity programme. They consider it important to have sufficient employment places in this field after completing the training. Regarding the scope of practice, medical students who prefer general practice want to have long-term and close relationships with their patients and patients’ family members. Continuity of care and prevention is an important aspect in their future work and they want to support patients in managing their illnesses and mental health problems. Medical students who prefer general practice describe interest in regular, preferably daytime working hours. They want to have flexible working hours so they could have good opportunities to match work and private life.

After combining the factors from model 1 and 2, the correlation between being a woman and preferring general practice disappeared (see model 3). However, the importance of other factors identified in the initial models persisted, affirming their relevance in influencing medical students’ career intentions. Despite not being significant in model 1, the factor of coming from a rural area was revisited in model 4 alongside other factors. This inclusion led to the loss of significance for factors such as being a woman and being from Flanders. Model 4 highlighted the following predictors for preferring general practice: being a sixth-year medical student, coming from a rural area, and having GP role models. Additionally, considering factors such as postgraduate training opportunities, the scope of practice for general practice, and preferred working conditions remained crucial. The results of the logistic regression analyses are presented in Supplementary Table 4.

## Discussion

### Summary

Our study focused on describing the factors influencing the career intentions of medical students in Flanders (Belgium), Estonia, and Hungary. The aim was to create models to describe medical students who prefer a career in general practice and what they consider important in their future specialty.

The preferred specialty choices among the medical students participating in this study were surgical specialties, internal medicine and paediatric specialties, emergency medicine and anaesthesiology. There were differences between males and females with regard to specialty choices. The study found 18.5% of the medical students who participated are considering choosing general practice as their first or second choice.

We created four different models to describe medical students who prefer a career in general practice. Factors related to medical students and the curriculum that predicted the career intention of general practice in different combinations were being a woman, being a medical student from Flanders, being a sixth-year medical student, coming from a rural area, and having GP role models.

Medical students who prefer a career in general practice consider it important for the postgraduate training to have enough placements close to their place of residence. They also favour a postgraduate training with shorter duration and a lower intensity without complex admission requirements. In terms of their professional aspirations, these students prioritise the ability to establish long-term, close relationships with patients and their families, play a pivotal role in assisting patients with their physical and mental health challenges, emphasising the continuity of care and a preventive approach. Moreover, they seek regular, ideally daytime, working hours with the flexibility to adjust their schedules, highlighting the importance of maintaining a work–life balance. This preference profile suggests that medical students who are drawn to general practice are looking for a career path that not only allows for meaningful patient interactions but also supports a balanced lifestyle. Addressing these preferences in postgraduate training programmes and employment opportunities could enhance the appeal of a career in general practice and potentially leading to an increase in the number of medical students choosing this path.

### Strengths and limitations

To conduct this study we took into consideration previous research, we used a questionnaire that was employed in previous studies^20,21^ and conducted multivariable statistical analysis. We invited medical students from all the medical faculties in Flanders (Belgium), Estonia, and Hungary to participate in this study and thereby we were able to collect a relatively large sample. In this questionnaire we were able to present to medical students a wide range of factors and to consider these alongside demographic characteristics, rather than focusing on a narrow range of factors or characteristics. We also included medical students from all the study years and focused on exploring medical students’ career intentions and not their actual choice of specialty.

Although invitations to participate in this study were sent via universities’ official mailing lists, it was difficult to assess the actual reach. Since we did not ask at which university the students are studying, we do not have information on how many students participated from each university. The response rate varied between countries being the highest in Estonia and lowest in Hungary. In Estonia the questionnaire was sent to students right before the declaration of COVID-19 pandemic compared with Flanders and Hungary. Estonia’s advantage could also be the existence of only one medical faculty. As our questionnaire was comprehensive, it took at least 15 minutes to fill it out and therefore a number of students left the questionnaire unfinished and so were not included in the analysis.

Owing to the voluntary participation, a selection bias in favour of students more interested in this topic cannot be excluded. As the study is based on a cross-sectional survey, we cannot conclude causality in the analysis. Generalisation of our findings is limited.

### Comparison with existing literature

With regard to the factors related to GP career intention, our findings are consistent with previous evidence. Different studies have reported various proportions of medical students interested in general practice starting as low as 3% in Greece,^23^ 7% among students in Germany,^24^ 14% in the US,^25^ and as high as 29% among last year medical students at the University of Aberdeen.^26^ There is insufficient data on how many GPs are needed to ensure adequate availability of primary care services. According to OECD, in 2021 across OECD countries GPs represented on average 23% of all physicians.^2^ We do know that medical students’ low interest in general practice predicts their final choice of specialty and that students tend not to switch into general practice if it was not considered during the medical studies.^27^

We found that medical students from Flanders were more likely to consider a career in general practice compared with students in Estonia and Hungary. This discrepancy cannot be explained only by the differences in the strength of primary care in these countries, as based on Kringos *et al* both Estonian and Belgian primary care systems were rated strong.^3^ Some factors explaining this can be differences in the economic conditions of primary care and remuneration of GPs in these countries,^2,28^ as GPs in Belgium earn much more than in Estonia and Hungary.^3^ Based on OECD, Belgium also has the highest proportion of GPs (37%) compared with Estonia (23%) and Hungary (12%).^2^

Although some studies have found a strong correlation between being female and preferring a career in general practice,^5,17,29^ other studies^18,30,31^ similarly to ours, have described the disappearance of this correlation once factors characterising the future specialty were included. It shows that gender’s role is contextual: initially perceived as significant, gender’s influence on career intentions fades when analysed in conjunction with a broader set of variables, suggesting that its impact is interrelated with other factors. Previous research also suggests that any gender effect may be influenced by or owing to gender differences in work-related values and motivation.^9,32^

Prior studies have demonstrated that the decision to pursue a career in general practice often happens later during the studies,^33^ in an advanced age^17,24,34–36^ or in early postgraduate training.^37^ We found that sixth-year medical students showed the most interest in a GP career, but did not see any correlation based on age alone. This could indicate several important trends and implications in medical education. First, by their final year, medical students have been exposed to a wide range of specialties. Second, final-year students might have a better understanding of the critical role GPs play in preventive care, early detection of diseases, and the management of chronic conditions, making the role more appealing. Also, exposure to general practice through clinical rotations happens often in the final years of education^38^ and can have considerable influence on students’ perceptions, especially if they have positive experiences with mentorship. A study in Denmark also showed that the impact of specialty on work–life balance becomes a more important consideration in the later stage in studies.^30^ Medical students also often believe that a career in general practice could provide a better quality of life compared with the hospital specialties.^9–12,19,39,40^

In all our models the strongest predictor of interest in general practice was having GP role models. We know from previous studies that the existence of positive GP role models and having positive experiences in general practice,^6,7,14,41,42^ especially at the early stage of medical studies,^16^ were linked to an increased likelihood to choose a career in general practice. At the same time, negative experiences influenced the choice in the other direction.^41,42^

Regarding the factors related to certain specialties, our results are comparable with previous studies. A study in the UK also described the increasing importance of the availability of career posts as a factor influencing career choices among physicians.^39^ Consistent with previous studies, medical students who are considering a career in general practice value continuity of care^26,31,38,39^ and long-term and close relationships with their patients and their family members.^26^ There is also overwhelming evidence that medical students and newly qualified physicians consider achieving work–life balance and having flexible working conditions an increasingly important factor when considering future specialty,^9–12,19,38,39^ influencing even more strongly the choice in favour of general practice.^5,39,40^ Interestingly, some studies have reported aversion of or disappointment with hospital environment and highly specialised medicine as one of the reasons for choosing a career in general practice.^26,31^ Many students also prefer general practice because of the opportunity to work in and being part of a team.^26,31^ It may be that, since GPs in Belgium, Estonia, and Hungary work mainly in solo practices,^3^ working in teams was seen by our participants as a characteristic of hospital specialties.

### Implications for research and practice

This study enhances our understanding of the factors influencing medical students’ intentions to pursue a career in general practice. The results of this study reflect the complexity of making career choices, highlighting how the importance of certain factors can change with the introduction or combination of others. Despite the changes in the significance of some factors, the consistent importance of elements such as being advanced in the studies, having GP role models, and certain attitudes towards postgraduate training and work conditions, suggest a set of key factors for preferring a career in general practice. Additionally, we found a significantly lower interest in a career in general practice among medical students from Estonia and Hungary compared with Flanders. Although interest in a specific specialty does not always reflect the actual choice, a more in-depth study of the reasons for these differences between countries can provide additional information about what measures could be implemented in other countries to increase students’ interest in general practice.

The most important predictor of career intention in general practice was having GP role models. Therefore, it is very important that the undergraduate curriculums include opportunities for medical students to observe and interact with GPs as early as possible, including through shadowing, clinical rotations, and clinical case discussions led by GPs who are not only skilled but also reflect values that could inspire students. Further research, including qualitative research into when and where medical students come into contact with role models and what qualities they value in their role models, could give us better understanding on how medical faculties and GP organisations can support GPs to be better advocates for their specialty.

This study underscores the importance of promoting primary care as a rewarding career path, through both curriculum development and policy measures aimed at supporting both current and future GPs. Medical faculties might need to enhance their focus on general practice teaching and exposure training, ensuring that students are well-prepared for and informed about the opportunities in this field. Understanding the importance and supporting these developments can be crucial for addressing the future needs of healthcare systems worldwide.

## Data

The data set is available on request.
